# Optimization of a Natural-Deep-Eutectic-Solvent-Based Dispersive Liquid–Liquid Microextraction Method for the Multi-Target Determination of Emerging Contaminants in Wastewater

**DOI:** 10.3390/molecules30142988

**Published:** 2025-07-16

**Authors:** Beatriz Gómez-Nieto, Antigoni Konomi, Georgios Gkotsis, Maria-Christina Nika, Nikolaos S. Thomaidis

**Affiliations:** 1 Departamento de Química Analítica y Análisis Instrumental, Facultad de Ciencias, Universidad Autónoma de Madrid, Avda. Francisco Tomás y Valiente, 7, 28049 Madrid, Spain; beatriz.gomez@uam.es; 2 Laboratory of Analytical Chemistry, Department of Chemistry, National and Kapodistrian University of Athens, Panepistimiopolis Zografou, 15771 Athens, Greece; antigoniknm@chem.uoa.gr (A.K.); geogkotsis@chem.uoa.gr (G.G.); nikamar@chem.uoa.gr (M.-C.N.)

**Keywords:** hydrophobic NADES, microextraction, emerging contaminants, wastewater, green analytical chemistry, UHPLC-QTOF-MS

## Abstract

The widespread discharge of industrial and urban waste has led to significant increases in the environmental concentrations of numerous chemical substances. This work presents the development of a simple and environmentally friendly dispersive liquid–liquid microextraction (DLLME) method based on a hydrophobic natural deep eutectic solvent (NADES) for the determination of selected compounds from benzotriazole, benzothiazole, paraben, and UV filter families in wastewater samples. Of the twelve NADES formulations evaluated, those composed of a 4:1 molar ratio of thymol and menthol presented the highest extraction efficiencies. The influence of key experimental variables such as the pH of the aqueous sample, the ratio of NADES phase to sample volume, and the extraction time on the extraction efficiency was investigated using a multivariate optimization. Under optimal conditions, relative standard deviations below 15% and recoveries for spiked wastewater samples ranged between 82 and 108%, demonstrating the suitability of the method for routine water-quality monitoring. The sustainability and practicality of the developed method was evaluated using the assessment tools ChlorTox, AGREEprep, AGRRE, and BAGI, obtaining scores of 0.005 g in the NADES-DLLME method, 0.70, 0.52, and 72.5, respectively, demonstrating that the method is green and reliable.

## 1. Introduction

The pollution of water resources from industrial, agricultural, and/or urban activities has increased in recent decades due to the growing production and use of chemical compounds such as antibiotics, pesticides, preservatives, and UV filters that are discharged into the environment without proper controls. Many of these substances are classified as emerging contaminants (ECs) due to their increasing presence in the environment, persistence, and endocrine-disrupting properties, raising increasing concerns about their potential ecological effects and risks to human health [1,2,3,4,5,6]. Wastewater treatment plants (WWTPs) are a major source of entry of these contaminants in aquatic ecosystems, as conventional treatment processes are often insufficient for their complete removal [6,7]. Therefore, the development of accurate analytical methods for monitoring the presence of these substances in water resources and WWTP effluents is crucial to ensure the protection of both aquatic biota and human health.

Determining the presence of ECs in environmental samples is a complex task due to the large number of ECs, the typically low concentrations in the environment, and the complexity of the samples. The detection and quantification of organic ECs is commonly carried out using liquid chromatography (LC) and gas chromatography (GC) coupled to single or tandem mass spectrometry (MS or MS/MS), using low- or high-resolution mass analyzers, due to the high sensitivity and selectivity provided by these techniques [8]. In routine analysis, many multi-class methods have been developed to increase sample throughput. These methods are designed to detect and determine a great number of ECs from different chemical families using only a single analytical procedure and measurement run, reducing the analysis time and cost while maximizing data collection [9,10,11,12,13].

Regardless of the quantification technique used, sample preparation is a crucial step to extract and preconcentrate the analytes of interest from matrix interferences. Several extraction and clean-up techniques have been developed for the determination of ECs. Among them, solid-phase extraction (SPE) and liquid–liquid extraction (LLE) are the most widely reported in the literature [3,12,13,14,15,16]. In multi-class analysis, the most commonly used strategy is SPE using mixed sorbent cartridges, such as layered “mixed bed” cartridges, to cover a wide polarity range of analytes. However, these sample preparation methods are often tedious, time-consuming, and require large amounts of adsorbents and organic solvents, which can have a negative impact on the environment. To simplify these procedures and align them with the principles of green analytical chemistry (GAC) [17] and green sample preparation (GSP) [18], miniaturized versions of these techniques such as solid-phase microextraction (SPME) and dispersive liquid–liquid microextraction (DLLME) have gained significant attention in the past decade due to their high extraction efficiency in a relatively short time and their reduced use of adsorbents and/or organic solvents [14,19,20,21,22]. However, despite these advances in terms of sustainability, many microextraction techniques still use toxic organic solvents, making full compliance with GAC principles challenging. To address this limitation, recent research has focused on the use of more environmentally friendly solvents such as natural deep eutectic solvents (NADESs) in sample preparation procedures to reduce the use of organic solvents [23,24]. NADESs are a novel class of solvents derived from natural substances such as fatty acids, alcohols, carbohydrates, and/or sugars that are characterized by their low toxicity and high biodegradability. These solvents are binary or ternary mixtures composed of a hydrogen bond acceptor (HBA) and one or more hydrogen bond donors (HBD), compounds that, when mixed in the appropriate molar ratio, form a homogeneous liquid with a melting point lower than that of their individual components. This process is entirely atom-economic, generating no waste and requiring no additional purification steps. In addition, NADESs are distinguished by their simple and economical synthesis and the versatility and adaptability of their physicochemical properties, including their polarity, due to the wide range of possible HBA and HBD combinations [25,26,27,28]. NADESs have demonstrated a high capacity for extracting both organic and inorganic compounds in various applications, enabling extractions from solid and liquid environmental samples [29,30,31,32]. However, most of the NADES-based methods developed for extracting organic pollutants from water samples are tailored to specific chemical families, limiting their applicability and making multi-class analyses time-consuming and expensive. For example, Y. Lu et al. [33] reported a method for the extraction of six UV filters in surface and bathing waters, J. Cao et al. [34] developed a procedure for extracting parabens and their metabolite from surface water samples, while A. Yi-Ting et al. [35] optimized a DES-based microextraction method for determining benzotriazole and benzothiazole derivatives in surface water samples.

The aim of this work was to optimize a simple and green sample preparation method for the simultaneous preconcentration of different families of industrial chemicals (benzotriazoles, benzothiazoles, parabens, and UV filters) using hydrophobic NADESs as the extraction medium. To the best of the authors’ knowledge, this is the first NADES-based method designed for multi-class analysis of these emerging contaminants.

## 2. Results

### 2.1. Evaluation and Selection of the NADES Extractant Phase

**Figure 1:**
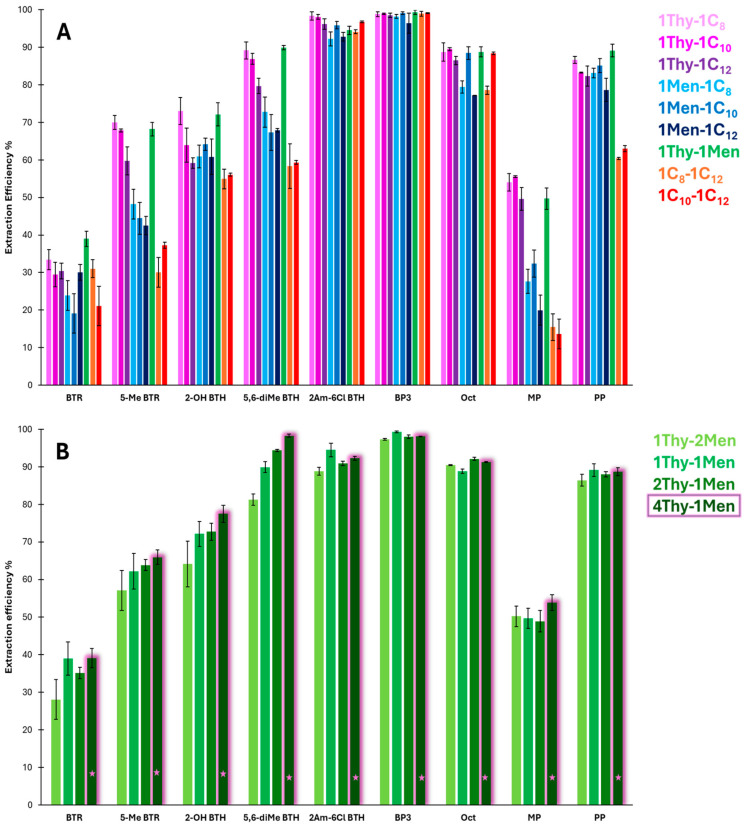
(**A**) Extraction efficiency for the different analytes and NADESs evaluated (n = 3). (**B**) Extraction efficiency for thymol-menthol based NADES. The selected NADES is highlighted with a pink star symbol.

The extraction solvent plays a crucial role in the efficiency of the DLLME process. An ideal extractor for organic contaminants in aqueous samples should be dispersible in aqueous media and possess low viscosity to enhance the mass transfer of the analyte. In this study, the extraction efficiency (EE, %) of hydrophobic NADESs composed of poorly water-soluble monoterpenes and long-chain fatty acids [26,36,37] for the simultaneous extraction and preconcentration of the nine target analytes selected (see Section 3.1) from the four contaminant families studied in this work (benzotriazoles (BTRs), benzothiazoles (BTHs), UV filters, and parabens) were evaluated. To this end, 5.00 mL of aqueous standards solutions and 100 µL of the corresponding NADES were manually mixed for 2.5 min. These initial conditions were selected considering the typical parameter ranges reported in DLLME-based methods. To select the more appropriate components of the NADESs, initial studies were carried out using NADESs prepared in a 1:1 molar ratio (see detailed composition of NADESs included in Section 3.2). The EE% of each NADES was calculated as the ratio between the amount of analyte extracted into the NADES phase and the initial amount present in the aqueous standard solution. The EE% for each analyte and NADES is shown in Figure 1A. As can be seen in the figure, the EE% depended strongly on the NADES composition. In general, NADES formulations containing thymol and fatty acids with different chain lengths (NADESs 1Thy-1C_8_, 1Thy-1C_10_, or 1Thy-1C_12_) provided higher overall EE% than NADESs formulated with menthol and fatty acids (NADESs 1Men-1C_8_, 1Men-1C_10_, or 1Men-1C_12_). Since thymol differs structurally from menthol only by the presence of an aromatic ring instead of a saturated ring, these results suggest that the use of aromatic terpenes such as thymol in the preparation of NADESs could enhance the extraction of the target analytes mainly through pi–pi interactions between the aromatic rings of thymol and those of the analytes. In contrast, the NADESs composed solely of long-chain fatty acids (NADESs 1C_8_–1C_12_ and 1C_10_–1C_12_) showed the lowest overall EE%. This could be due to the limited partitioning of polar and moderate polar analytes into these hydrophobic NADESs and the absence of aromaticity in the NADES components, which reduce the affinity toward the analytes. In addition, the higher viscosity of menthol- and fatty-acid-based NADESs, particularly those containing longer chain lengths such as dodecanoic acid, could hinder phase dispersion and analyte diffusion [28,36,37,38], reducing the EE% in comparison to NADESs with shorter chain lengths.

Out of the NADESs with the higher EE%, the NADES composed of the combination of the monoterpenes thymol and menthol (1Thy-1Men) was selected as the most suitable for the following studies due to its high overall EE% and enhanced precision. To confirm the influence of thymol on the extraction capacity of this NADES and establish the optimal composition to carry out the simultaneous extraction of the nine analytes, NADESs with varying molar ratios of Thy-Men from 1:2 to 4:1 were also prepared and evaluated for the extraction process (Figure 1B). As can be seen in Figure 1B, the EE% of nearly all analytes increased as the amount of thymol in the NADES increased, obtaining the highest overall EE% using the NADES composed of a 4:1 molar ratio of thymol–menthol (4Thy-1Men). These results support the key role of thymol in enhancing the extraction of hydrophobic and aromatic compounds through pi–pi interactions and hydrogen bond formation with the analytes, which is consistent with the results found in previous works [39,40]. Moreover, the differences in EE% observed among thymol-based NADESs could also be attributed to the decrease in viscosity with increasing thymol content. In addition, the higher melting point of the 4Thy:1Men NADES may facilitates phase separation, minimizing analyte loss [41,42]. Consequently, the 4Thy-1Men NADES was selected as the medium for further extraction studies.

### 2.2. Multivariate Optimization of the NADES-DLLME Conditions

A two-step multivariate method was used to select and optimize the experimental conditions to perform the simultaneous extraction and preconcentration of the nine target analytes from aqueous standards. In the first step, the effect of the experimental variables on the EE% was evaluated using a two-level Plackett–Burman experimental design. The variables and the specific condition selected for each level (coded as −1 and +1) were pH of aqueous phase (level −1: pH 2; level +1: pH 9), NaCl content (level −1: 0.00 g L^−1^; level +1: 20.0 g L^−1^), agitation mode used for dispersion (level −1: manual agitation; level +1: ultrasonic bath agitation), extraction time (level −1: 1.0 min; level +1: 5.0 min), extraction temperature (level −1: 25 °C; level +1: 50 °C), aqueous phase volume (level −1: 5.00 mL aqueous phase; level +1: 10.0 mL aqueous phase), and centrifugation time (level −1: 3.0 min; level +1: 5.0 min). All extraction studies were carried out using aqueous standards and 100 µL of the 4Thy-1Men NADES. The overall response (OR) obtained from the individual EE found for each analyte in the corresponding experiment was used as the analytical response of the system (see Equation (1)).

(1) $$\mathrm{OR}=\sqrt[{9}]{{EE}_{BTR}\;\times \;{EE}_{5MeBTR}\;\times \;{EE}_{5.6diMeBTR}\;\times \;{EE}_{2OHBTH}\;\times \;{EE}_{2Am,6ClBTH}\;{\times \;EE}_{BP3}\;\times \;{EE}_{OCT}\;\times \;{EE}_{MP}\;\times \;{EE}_{PP}}$$

**Figure 2:**
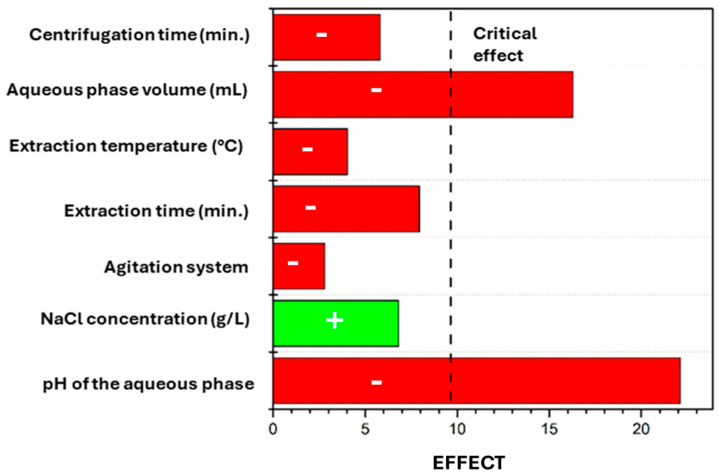
Effects of the variables obtained in the Plackett–Burman screening step. Positive effects: green; negative effects: red.

A more detailed description of the performed Plackett–Burman experimental design and the data evaluation procedure is included in Section S1.1 of the Supplemental Material (SM). Figure 2 presents the effect values (E_v_) obtained for each experimental variable and the critical effect (E_crit_). As can be seen in this figure, only two of the seven experimental variables evaluated—the pH and the volume of the aqueous phase used in the extraction—seem to exert a significant effect on the EE% of the analytes, since the absolute values of the effects for these variables are higher than the value of the calculated critical effect (∣E_V_∣ ≥ E_crit_). Therefore, these variables were selected to be optimized in the second step of the multivariate optimization process. The experimental conditions for variables with no significant effect were selected considering the sign of their effects. For the variables with a negative effect value (the agitation mode, the extraction time and temperature, and the centrifugation time) the conditions set for the −1 level of the Plackett–Burman experiments were selected, while for the variable with a positive effect value (NaCl concentration in the aqueous phase), the fixed condition for the +1 level was chosen. Thus, the following conditions were set for further studies: manual agitation for 1 min at 25 °C (room temperature), addition of a concentration of NaCl in the aqueous phase of 20 g L^−1^, and centrifugation for 3.0 min (at 3000 rpm).

**Figure 3:**
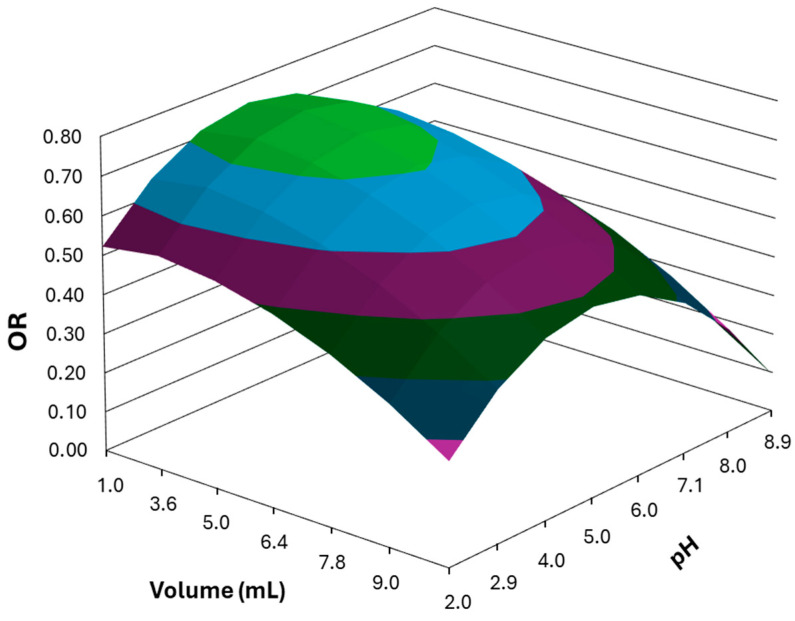
Response surface obtained in the optimization of aqueous phase volume (mL) and pH for the microextraction of the target analytes.

The aqueous phase pH and volume were optimized using a five-level central component design (CCD) and response surface model (RSM). The influence of these variables was examined between 1.00 and 9.00 mL for aqueous phase volume and between 2.0 and 8.0 for aqueous phase pH, using only 11 experiments (22 experiments to evaluate first-order effects; 2 × 2 experiments to evaluate second-order effects; and three replicates of the central point experiment). All the extractions were performed using 100 µL of the 4Thy-1Men NADES and the experimental conditions previously established in the screening step. The OR was used as the analytical response of the system (see Equation (1)). A more detailed description of the CCD experiments and the data evaluation procedure used to assess the quality of the model and the construction of the RSM can be found in Section S1.2 of the Supplemental Material. From the obtained mathematical model (see Equation (S6)), the response surface plot shown in Figure 3 was constructed. The OR of the system was found to be maximal and stable within the range of aqueous phase volumes of 1.00 to 5.00 mL and pH values of 4.0 to 6.0. To achieve the highest enrichment factor, an aqueous phase volume of 5.00 mL was established as optimal. On the other hand, the pH of the aqueous phase was set at approximately 5.0, although setting an exact pH value is not critical if it remains within the range of 4.0 to 6.0. The final conditions for the microextraction procedure are summarized in Section 3.4.

### 2.3. Calibration and Analytical Figures of Merit

To assess the analytical performance of the method, the following parameters were evaluated: linearity, limits of detection and quantification (LODs and LOQs), enrichment factors (EFs), sensitivity enhancement factors (SEFs), and repeatability. The results for each parameter and analyte are summarized in Table 1. To avoid interference from NADES components, the calibration curves were constructed using the so-called NADES-matched calibration curves [35] using multi-analyte standard solutions prepared in a NADES–MeOH solution containing concentrations ranging from 2.00 to 100 ng mL^−1^ and a fixed concentration of diuron-d_6_. Linear correlations with an r^2^ greater than 0.996 were obtained for all calibration curves, demonstrating good linearity within the tested range. The instrumental LODs and LOQs of each analyte were estimated by measuring the signal-to-noise ratio (S/N) of a multi-analyte standard solution of 10.0 ng mL^−1^ 3 and 10 times, respectively (see Table S4). The enrichment factors (EFs) of the DLLME method were calculated as the ratio between the concentration found in the NADES extract after DLLME of an aqueous standard solution of 2.00 ng mL^−1^ and the initial analyte in the aqueous phase. The LODs and LOQs of the method were determined by considering the corresponding instrumental limits, the EFs found for each analyte, and the dilution ratio of the NADES extract medium before the UHPLC-QTOF-MS measurement. The obtained LOD and LOQ values are comparable to those reported in other NADES-DLLME-LS-MS methods [11,35]. In contrast, these values are approximately ten times higher than those achieved with conventional SPE methods before LS-MS analysis [13,16]. However, it should be noted that the methods described in these references use larger sample volumes and require significantly longer extraction and preconcentration times.

The sensitivity enhancement factors (SEFs) were calculated by dividing the instrumental LODs or LOQs by those found for the method. The precision of the method was evaluated at two concentration levels by examining the relative standard deviation (RSD%) of triplicate extractions of multicomponent aqueous standards of 1.00 and 4.00 ng mL^−1^ on the same day. For the multicomponent standard containing 1.00 ng mL^−1^ (level 1), RSD values ranging from 5.1% to 13.5% were found for all analytes, while for the standard containing 4.00 ng mL^−1^ (level 2), RSD values lower than 7.3% were achieved, demonstrating the good repeatability of the proposed method.

### 2.4. Evaluation of the Sustainability, Practicality, and Applicability of the Method

The environmental impact of the developed method was assessed using three complementary sustainability metrics: the Chloroform Toxicity Estimation Scale (ChlorTox), the Analytical GREEnness Preparation (AGREEprep) metric, and the Analytical GREEnness (AGREE) metric [43,44,45,46]. In addition, the Blue Applicability Grade Index (BAGI) was used to identify the weak and strong points of the method in terms of practicality and applicability [47]. The selected greenness metrics used in this work offer different perspectives on the sustainability of the method: ChlorTox estimates the health and environmental hazards associated with specific chemicals used in the method; AGREEprep assesses the greenness of the sample preparation method based on the 10 principles of GSP; and AGREE assesses the impact of the entire analytical method based on the 12 principles of GAC. The overall scores and/or pictograms obtained for each metric tool are shown in Table 2. The specific criteria and individual values used for the calculation of the final scores of each tool are included in Section S3 of Supplementary Materials.

The ChlorTox score was calculated for both the DLLME sample treatment method and the complete DLLME-HPLC-QTOF analytical method. This metric compares the potential hazards of a substance with those of chloroform to obtain the so-called chloroform-equivalent mass. To determine the toxicity of the substances used in the method, the weighted number of hazards (WNH) was calculated for each reagent; this value was normalized using the WNH of chloroform and multiplied by the mass of reagent used in a single analysis (see individual reagents values in Table S5). The final ChlorTox score corresponds to the sum of the chloroform-equivalent mass values for all reagents involved in the method. As shown in Table 2, the developed NADES-based DLLME method presents a very low ChlorTox mass value of 0.005 g, indicating the minimal chemical hazard associated with the sample preparation method. However, this value increases substantially to 6 g when the full analytical method is considered, due to the large volume of mobile phases composed of MeOH used for chromatography separation.

The AGREEprep and AGREE tools evaluate the sustainability of the sample treatment method and the entire analytical method, respectively. The results of both tools are visually presented in circular pictograms divided into 10 sections (one for each GSP principle) in AGREEprep or 12 sections (one for each GAC principle) in AGREE, to which a numerical score, also associated with a color scale, ranging from 1 (green) to 0 (red), is assigned based on the environmental impact of the method conditions with respect to the corresponding principle. The final score of the analytical procedure is displayed in the center of the pictogram. The final AGREEprep and AGREE scores of the developed method were 0.70 and 0.52, respectively (see Table 2). The higher AGREEprep score achieved in the sample preparation evaluation, highlighted in green in the pictogram, is mainly due to the use of sustainable reagents, a factor that has a direct impact on the toxicity and operator safety criteria, as well as the reduced time and energy requirements of the extraction process. On the other hand, the most negatively affected criterion in both AGREEprep and AGREE metrics, highlighted in red in the respective pictograms, is related to the location where both the sample pretreatment and instrumental analysis are performed (criterion 1), as these steps are carried out off-line in the laboratory. Other criteria that did not achieve green ratings in the AGREE evaluation are those related to energy consumption and waste generation. Despite these limitations, the overall scores in both assessment tools exceed 0.50, suggesting that the method has a moderate environmental impact and complies with most of the GAC principles.

Finally, the practicality and applicability of the developed method were evaluated using the BAGI metric. This tool considers 10 criteria or attributes related to the method characteristics in the evaluation, from the type of analysis, instrumental technique, and number of analytes simultaneously determined to the type of sample preparation and the necessity of its use to meet the required sensitivity to analyze real samples. The result is displayed as a pictogram with the overall score in the center. The score of each attribute is indicated by a blue color scale, in which dark blue and light blue denote high and low compliance with the criteria, respectively. The specific criteria evaluated and the corresponding values assigned to the developed method are included in Table S7. According to N. Manousi et al. [47], a method could be considered applicable and practical when the overall BAGI score is higher than 60. As can be seen in Table 2, the BAGI score obtained for the method was 72.5, confirming its high level of applicability and practicality. This score could be further improved by extending the method to include additional target analytes in the analytical protocol.

### 2.5. Application and Validation

The developed method was applied to determine the concentrations of the nine target contaminants in four influent wastewater (WW) samples collected from Greece. For each sample, three aliquots of 5.00 mL were subjected to the DLLME procedure described in Section 3.4 and subsequently measured using the UHPLC-QTOF-MS measurement conditions specified in Section 3.5. The observed concentrations for each analyte in the samples are presented in Table 3. Of the analyzed benzotriazoles and benzothiazoles, BTR was the most frequently detected compound, with concentrations ranging from 0.32 to 0.84 ng mL^−1^. In contrast, 5-Me BTR and 2-OH BTH were only quantified in WW-1 (0.35 and 0.82 ng mL^−1^, respectively), while 5,6-diMe BTR and 2Am-6Cl BTH were not detected in any sample, suggesting limited occurrence or effective removal during wastewater treatment. Among the UV filter compounds evaluated, OCT was detected in all samples (0.51–0.91 ng mL^−1^), while BP3 was only detected in samples from the same city (WW-1 and WW-2) at similar concentrations. In addition, parabens (MP or PP) were not detected in any of the samples (MP < 0.088 ng mL^−1^; PP < 0.024 ng mL^−1^), suggesting either their effective removal in the WWTPs or low usage in the sampled areas. Similar concentrations of benzotriazoles, benzotriazoles, and UV filters in wastewater samples have been reported previous studies [13,16,48].

To assess the validity of the developed method, the sample WW-1 was spiked with 0.200 ng mL^−1^, subjected to the NADES-based DLLME protocol, and analyzed using the UHPLC-QTOF-MS method. The calculated concentrations for each analyte in the fortified sample and the obtained recoveries are presented in Table S8 of the Supplemental Material. Good recoveries ranging from 82% to 108% were achieved, indicating that the water sample matrix had no significant influence on the extraction efficiency of the analytes.

## 3. Materials and Methods

### 3.1. Reagents and Standard Solutions

As representative analytes of the chemical families benzotriazoles, benzothiazoles, parabens, and UV filters, the following compounds were selected: 1-H-benzotriazole (BTR), 5-methyl-benzotriazole (5Me-BTR), 5,6-dimethyl-benzotriazole (5,6-diMe-BTR), 2-hydroxy-benzothiazole (2OH-BTH), 2-amino-6-chloro-benzothiazole (2Am-6Cl-BTH), benzophenone-3 (BP3), octocrylene (Oct), methylparaben (MP), and propylparaben (PP). The chemical structures and properties of the analytes are included in Table S9 of Supplemental Material. The selection of these compounds was based on their increasing prevalence in aquatic environments and potential environmental risks.

Individual standard solutions of the target analytes at a concentration of approximately 1000 mg L^−1^ were prepared by dissolving each compound in methanol (MeOH) and were stored in a freezer for further use. Mixed working standard solutions were prepared by suitable dilution of the 1000 mg L^−1^ standards. Diuron-d6 (Sigma Aldrich (Steinheim, Germany)) was used as an internal standard in UHPLC-QTOF-MS measurements. For the preparation of the evaluated NADESs, DL-menthol, thymol, octanoic acid, decanoic acid, and dodecanoic acid with a purity of 99% (Sigma Aldrich, Germany) were used. LC-MS-grade solvents and reagents: MeOH (Fischer Scientific (Loughborough, UK)), formic acid (CARLO ERBA Reagents S.A.S. (Barcelona, Spain)), ammonium acetate (Sigma Aldrich (Steinheim, Germany)), and ammonium formate (Sigma Aldrich (Steinheim, Germany)) were used to prepare the mobile phase for the chromatographic separation. Ultrapure water with a resistivity of at least 18.2 MΩ cm obtained from a water purification system (Millipore, Direct-Q UV, Bedford, MA, USA) was used throughout.

### 3.2. NADES Preparation

Hydrophobic NADESs with varying compositions were prepared by combining different monoterpenes (menthol (Men) and/or thymol (Thy)) and fatty acids with different carbon chain lengths (octanoic acid (C_8_), decanoic acid (C_10_), and/or dodecanoic acid (C_12_)) as HBA and/or HBD. The composition and molar ratios of the NADESs prepared is shown in Table 4. It is important to note that although these solvents are referred to as NADESs throughout this work, not all mixtures were prepared at their deep eutectic molar ratios or showed an ideal behavior [26,28,36,37,41,42].

The preparation of the NADESs was achieved by heating and stirring the appropriate amount of the individual components that form the NADES at approximately 400–500 rpm and 50 °C using a heating plate with a magnetic stirrer until a clear and transparent liquid was formed (about 10 min). The formed NADES was then cooled under constant stirring and stored at room temperature until use. The physicochemical properties of the prepared NADESs, including their viscosity, thermal behavior, and hydrogen-bonding interactions, have been previously studied and characterized in the literature [26,28,36,37,41,42]. The melting points and viscosities reported are included in Table 4.

### 3.3. Instrumentation

The chromatographic measurements were carried out using an ultrahigh-performance liquid chromatography (UHPLC) system, with an HPG-3400 pump (Dionex UltiMate 3000 RSLC, Thermo Fisher Scientific, Dreieich, Germany), coupled to a QTOF-MS spectrometer (Maxis Impact, Bruker Daltonics, Bremen, Germany). Post-acquisition data treatment was carried out with DataAnalysis 5.1. software (Bruker Daltonics, Germany). The standards and other reagents were weighed using an analytical balance (AB104-S Analytical Balance, METTLER TOLEDO, Columbus, OH, USA) with an accuracy of ±0.01 mg. A pH meter (Q30d Digital single-channel multi-meter, HACH Greece, Athens, Greece) was used for the measurement and adjustment of the solution pH. For the preparation of the NADESs a heating plate with a magnetic stirrer was used. To accelerate the separation of the NADES phase from the aqueous phase after DLLME extraction, a centrifuge (Neya 16R, MOULAS Scientific, Chalandri, Greece) was used.

### 3.4. Samples and Microextraction Procedure

**Figure 4:**
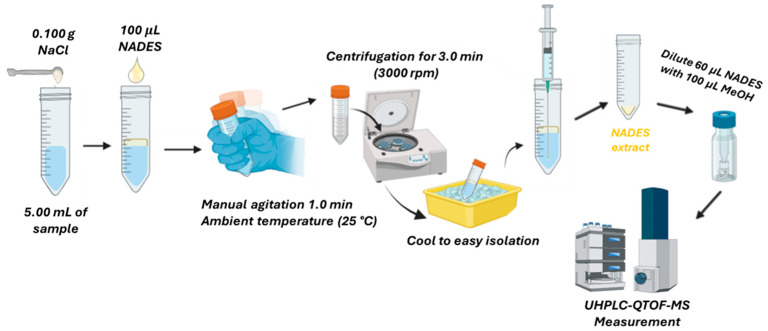
Schematic representation of the optimized NADES-based DLLME-UHPLC-QTOF-MS procedure. Created in BioRender.com.

The NADES-based DLLME method developed in this work was applied to analyze influent wastewater samples collected from WWTPs of two out of the four biggest cities in Greece. The samples were filtered with glass fiber filters of 0.7 µm and stored at 4 °C until analysis. The extraction procedure was performed as follows: 5.00 mL of wastewater sample was pipetted to a polyethylene centrifuge tube, and 0.100 g of NaCl was added to adjust the salinity of the sample to 20.0 g L^−1^. Then, 0.100 mL of NADES was added and mixed manually for 1.0 min. To isolate the NADES, the mixture was centrifuged for 3.0 min at 3000 rpm, cooled in the freezer until the NADES phase solidified (about 5.0 min), and the water phase was removed with a syringe with a long needle. For the analytical measurement of the extract phase, 60.0 µL of the isolated NADES was pipetted and diluted with 100 µL of MeOH containing the internal standard diuron-d_6_. This procedure was carried out in triplicate. Figure 4 includes a schematic representation of the NADES-DLLME-UHPLC-QTOF-MS procedure.

### 3.5. UHPLC-QTOF-MS Measurement Conditions

The chromatographic separation was carried out using an Acclaim RSLC C18 column (100 mm × 2.1 mm, 2.2 μm) from Thermo Fisher Scientific (Germany) thermostatted at 30 °C and the gradient elution program described in Table S10. The mobile phase used depends on the electrospray ionization mode used (positive or negative). The mobile phase composition in positive electrospray ionization mode (+ESI) consisted of (A) H_2_O:MeOH (90:10) with 5 mM ammonium formate and 0.01% formic acid, and (B) MeOH with 5 mM ammonium formate and 0.01% formic acid. For negative ionization mode (−ESI), the mobile phase consisted of (A) H_2_O:MeOH (90:10) with 5 mM ammonium acetate, and (B) MeOH with 5 mM ammonium acetate. An injection volume of 5 µL was used in all measurements. The QTOF-MS detector operated in +ESI (capillary voltage, 2500 V) for the determination of the studied benzotriazole, benzotriazole, and UV filter compounds and in −ESI (capillary voltage, 3500 V) for the determination of paraben compounds. In both ESI modes, the plate offset was set to 500 V, and the nebulizer gas flow, pressure, and temperature were set at 8 L min^−1^, 2 bar, and 200 °C, respectively. An external calibration was performed using a 10 mM sodium formate 1:1 water–isopropanol solution and a segment (0.1–0.25 min.) in every chromatogram. The retention times and the *m/z* fragments used for identification and quantification of each analyte are shown in Table 5.

## 4. Conclusions

This work presents a simple and rapid DLLME method for the simultaneous extraction and preconcentration of nine organic pollutants belonging to four different chemical families: BTRs, BTHs, UV filters, and parabens. Considerable efforts were devoted to minimizing the environmental impact of DLLME procedures by evaluating the extraction performance of hydrophobic NADESs formulated from terpenes and/or fatty acids. Among the twelve combinations tested, the NADES composed of thymol and menthol in a 4:1 molar ratio was identified as the most suitable, achieving an EE% ranging from 40 to 100% depending on the analyte hydrophobicity. Multivariate experimental designs were used to establish optimal extraction experimental conditions with a minimal number of experiments. The developed sample preparation procedure enables the extraction and preconcentration of the target analytes in just 1.0 min through manual agitation, eliminating the need for expensive or energy-intensive equipment. The sustainability of the developed NADES-DLLME method was supported by an AGREEprep score of 0.70, indicating compliance with most of the principles of GAC. The analytes in the NADES extracts were determined using UHPLC-QTOF-MS, achieving a 10- to 20-fold improvement in the LOQs of the method and RSD% values below 15%. The DLLME-UHPLC-QTOF-MS method was successfully applied to analyze wastewater samples obtaining recovery values between 82% and 108%, confirming the method accuracy for monitoring water pollutants. In our opinion, this study offers a novel contribution to the field of environmental analysis by integrating a cutting-edge analytical technique with environmentally friendly practices.

## Figures and Tables

**Table 1:** Analytical figures of merits of the optimized method.

Analyte	DLLME EFs	LOD ^a^/ng mL^−1^	LOQ ^a^/ng mL^−1^	SEFs	Precision ^b^/%	
					Level 1	Level 2
BTR	32 ± 3	0.058	0.19	12	11.3	9.0
5Me-BTR	36 ± 3	0.044	0.15	14	5.1	3.9
2OH-BTH	50 ± 6	0.070	0.23	19	13.2	7.3
5,6diMe-BTR	55 ± 4	0.048	0.16	19	12.0	5.2
2Am,6Cl-BTH	53 ± 2	0.016	0.052	19	8.2	2.2
BP3	49 ± 5	0.069	0.23	18	12.0	5.9
OCT	53 ± 2	0.071	0.24	19	6.8	4.1
MP	29 ± 2	0.088	0.26	11	13.5	6.2
PP	52 ± 4	0.024	0.072	19	10.4	6.6

^a^ DLLME-UHPLC-QTOF-MS method LODs and LOQs. Instrumental LODs and LOQs are included in Supplemental Material. ^b^ Aqueous standards concentrations were as follows: level 1: 1.00 ng mL^−1^; level 2: 4.00 ng mL^−1^.

**Table 2:** Scores and/or pictograms obtained using sustainability, practicality, and/or applicability used metric tools.

Metric Tool	Score/Pictogram
**ChlorTox**	DLLME: 0.005 g DLLME UHPL-QTOF-MS: 6 g
**AGREEprep** **1.** Sample preparation placement **2.** Hazardous materials **3.** Sustainability–renewability of materials **4.** Waste **5.** Size economy of the sample **6.** Sample throughput **7.** Integration and automation **8.** Energy consumption **9.** Post-sample preparation analysis **10.** Operator’s safety	*** 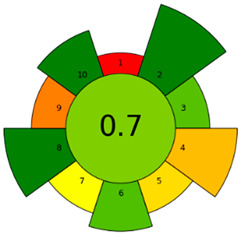 ***
**AGREE** **1.** Sample treatment placement **2.** Sample amount **3.** Device position **4.** Sample prep. stages **5.** Automation/minimization **6.** Derivatization **7.** Waste **8.** Analysis throughput **9.** Energy consumption **10.** Source of reagents **11.** Toxicity **12.** Operator’s safety	*** 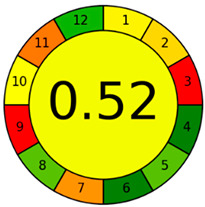 ***
**BAGI** **1.** Type of analysis **2.** Multi- or single-analyte determination **3.** Analytical technique **4.** Simultaneous sample preparation **5.** Sample pretreatment **6.** Samples analyzed per hour **7.** Reagents and materials **8.** Necessity of preconcentration **9.** Degree of automation **10.** Amount of sample	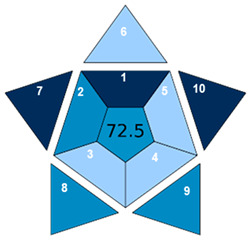

**Table 3:** Concentrations (mean ± standard deviation, n = 3) found for the target analytes in the analyzed wastewater samples using the optimized NADES-based DLLME-UHPLC-QTOF-MS method.

Analyte Concentration (ng mL^−1^)	Samples			
	WW-1	WW-2	WW-3	WW-4
BTR	0.32 ± 0.05	0.60 ± 0.05	0.52 ± 0.05	0.84 ± 0.09
5Me-BTR	0.35 ± 0.02	<0.044	<0.15	<0.15
2OH-BTH	0.82 ± 0.03	<0.070	<0.070	<0.070
5,6diMe-BTR	<0.048	<0.048	<0.048	<0.048
2Am,6Cl-BTH	<0.016	<0.016	<0.016	<0.016
BP3	0.57 ± 0.03	0.56 ± 0.06	<0.23	<0.23
OCT	0.91 ± 0.03	0.51 ± 0.02	0.53 ± 0.05	0.71 ± 0.03
MP	<0.088	<0.088	<0.088	<0.088
PP	<0.024	<0.024	<0.024	<0.024

**Table 4:** Evaluated NADES composition.

NADES	Components (HBA-HBD)	Molar Ratio	Melting Point (°C)	Viscosity (mPa s) ^a^
1Thy-1C_8_	Thymol–octanoic acid	1:1	11 [28]	7–10 [28]
Thy-1C_10_	Thymol–decanoic acid	1:1	15 [28]	10–15 [28]
1Thy-1C_12_	Thymol–dodecanoic acid	1:1	28 [28]	12 [28]
1Men-1C_8_	Menthol–octanoic acid	1:1	−5 [28]	12–20 [28]
1Men-1C_10_	Menthol–decanoic acid	1:1	7 [28]	15–25 [28]
1Men-1C_12_	Menthol–dodecanoic acid	1:1	23 [28]	21–28 [28]
1Thy-1Men	Thymol–menthol	1:1	−8 [41]	35 [42]
1C_8_–1C_12_	Octanoic acid–dodecanoic acid	1:1	21 [37]	7–9 [37]
1C_10_–1C_12_	Decanoic acid–dodecanoic acid	1:1	23 [37]	9–13 [37]
1Thy-2Men	Thymol–menthol	1:2	−5 [42]	41 [42]
2Thy-1Men	Thymol–menthol	2:1	9 [42]	26 [42]
4Thy-1Men	Thymol–menthol	4:1	28 [41]	-

^a^ Values for ambient temperature (between 20 and 30 °C).

**Table 5:** Retention times, precursor ion *m*/*z*, and ionization mode for the evaluated analytes by UHPLC-QTOF-MS.

Analyte	Retention Time (min)	Precursor ION *m*/*z*	Ionization Mode
Benzotriazole (BTR)	6.88	136.0215	+
5,6-dimethyl benzotriazole (5,6diMe-BTR)	6.76	148.0869	+
5-methyl benzotriazole (5Me-BTR)	5.83	134.0713	+
2-hydroxy benzothiazole (2OH-BTH)	6.53	152.0165	+
2-amino-6-Chloro Benzothiazole(2Am-6Cl-BTH)	8.00	184.9934	+
Octocrylene (Oct)	13.36	379.2380	+
Methylparaben (MP)	5.97	151.0400	−
Propylparaben (PP)	8.30	179.0714	−

## Data Availability

Data are contained within the article.

## Supplementary Materials

The following supporting information can be downloaded at: https://www.mdpi.com/article/10.3390/molecules30142988/s1. Table S1: Conditions of Plackett–Burman experiments and overall response (OR) obtained. Uncoded values selected for each variable are included in brackets. Table S2: Conditions of central composite design of experiments and overall response (OR) obtained. Uncoded values selected for each variable are included in brackets. Table S3: Results of statical parameters for Student’s *t* test of the fitted mathematical model. Table S4: Instrumental UHPLC-QTOF-MS limits of detection (LODs) and limits of quantification (LOQs). Table S5: WNH values and used mass in a single analysis for each reagent. Table S6: Criteria answers in AGREEprep and AGREE metrics. Table S7: Criteria answers in BAGI metric. Table S8: Concentrations (mean ± standard deviation, n = 3) and recoveries found for the target analytes in the fortified wastewater 1 (WW-1). Table S9: Chemical structures and properties of the analytes. Table S10: UHPLC-QTOF-MS gradient elution program.
