# Supplementary material for: Optimization of a Natural-Deep-Eutectic-Solvent-Based Dispersive Liquid–Liquid Microextraction Method for the Multi-Target Determination of Emerging Contaminants in Wastewater
Source: Molecules. 2025 Jul 16;30(14):2988. doi: 10.3390/molecules30142988 (PMC12298753; doi:10.3390/molecules30142988)
Supplement: Supplementary file 1 [file molecules-30-02988-s001.zip › molecules-3759817-supplementary.pdf]

Optimization of a Natural-Deep-Eutectic-Solvent-Based Dispersive Liquid–Liquid Microextraction Method for the Multi-Target Determination of Emerging Contaminants in Wastewater

Beatriz Gómez-Nieto [1,2], Antigoni Konomi [2], Georgios Gkotsis [2], Maria-Christina Nika [2] and Nikolaos S. Thomaidis [2,*]

[1] Departamento de Química Analítica y Análisis Instrumental, Facultad de Ciencias, Universidad Autónoma de Madrid, Avda. Francisco Tomás y Valiente, 7, 28049 Madrid, Spain; beatriz.gomez@uam.es
[2] Laboratory of Analytical Chemistry, Department of Chemistry, National and Kapodistrian University of Athens, Panepistimiopolis Zografou, 15771 Athens, Greece; antigoniknm@chem.uoa.gr (A.K.); geogkotsis@chem.uoa.gr (G.G.); nikamar@chem.uoa.gr (M.-C.N.)
* Correspondence: ntho@chem.uoa.gr

SECTION S1. MULTIVARIATE DESIGN OF EXPERIMENTS

Section S1.1. Screening step:

The Plackett-Burman design is a two-level screening method (low (-1) and high (+1)) that consists in performing N experiments (where N must be a multiple of 4) to evaluate N-1 experimental variables. Thus, the study of the seven experimental variables evaluated in this work on the extraction efficiency (EE) of the target analytes was carried out by performing only eight experiments. Table S1 shows the low (-1) and high (+1) values selected for each experiment and variable, as well as the overall response (OR) obtained. All the experiments were performed with 100 µL of the extracted NADES phase composed of 4Thymol: 1Menthol.

Table S1. Conditions of Plackett-Burman experiments and overall response (OR)obtained. Uncoded values selected for each variable are included in brackets.

Experiment	Variable							OR
	V_1	V_2	V_3	V_4	V_5	V_6	V_7	
1	+1<br>(9)	+1<br>(20)	+1<br>(UA)	+1<br>(5)	+1<br>(50)	+1<br>(10.0)	+1<br>(5)	16
2	+1<br>(9)	-1<br>(0)	-1<br>(M)	+1<br>(5)	-1<br>(25)	-1<br>(5.0)	+1<br>(5)	47
3	+1<br>(9)	-1<br>(0)	-1<br>(M)	-1<br>(1)	+1<br>(50)	+1<br>(10.0)	-1<br>(3)	35
4	+1<br>(9)	+1<br>(20)	+1<br>(UA)	-1<br>(1)	-1<br>(25)	-1<br>(5.0)	-1<br>(3)	26
5	-1<br>(2)	+1<br>(20)	-1<br>(M)	-1<br>(1)	-1<br>(25)	+1<br>(10.0)	+1<br>(5)	68
6	-1<br>(2)	-1<br>(0)	+1<br>(UA)	-1<br>(1)	+1<br>(50)	-1<br>(5.0)	+1<br>(5)	51
7	-1<br>(2)	-1<br>(0)	+1<br>(UA)	+1<br>(5)	-1<br>(25)	+1<br>(10.0)	-1<br>(3)	43
8	-1<br>(2)	+1<br>(20)	-1<br>(M)	+1<br>(5)	+1<br>(50)	-1<br>(5.0)	-1<br>(3)	51

V_1: pH of aqueous phase; **V_2:** NaCl content (g/L); **V_3:** Agitation mode (UA= Ultrasonic bath; M= manual);
V_4: Extraction time (min.); **V_5:** Extraction temperature (°C); **V_6:** Aqueous phase volume (mL)
V_7: Centrifugation time (min.)

The effect of each variable (E_V) on the OR was obtained according to Eq. S1, where ΣY (+1) and ΣY (−1) are the sum of the OR values for those experiments where the variable V_x is at the high (+) level or the low (-) level, respectively, and N is the number of performed experiments.

$$E_V = \frac{\sum Y(+) - \sum Y(-)}{N/2} \qquad \textbf{(Eq. S1)}$$

The critical effect value (E_{crit}) was calculated using the modified algorithm of Dong (DONG-60%), according to Eq.S2, where t_{crit} is the tabulate t-student value for the total experimental variables evaluated for a significance level of 0.05 and $\sqrt{\frac{\sum E_{v-60\%}{}^2}{m}}$ is the standard error, calculated using only the 60% the effects with the lowest E_V absolute values.

$$E_{crit} = t_{crit}\sqrt{\frac{\sum E_{v-60\%}{}^2}{m}} \qquad \textbf{(Eq. S2)}$$

<u>Section S1.2. Optimization step:</u>

A five-level Central Composite Design (CCD) was used to optimize the experimental variables with critical effect on the extraction efficiency. The Central Component Design (CCD) is a multivariate design of experiments that facilitates the determination of the interrelationship between the experimental variables and the establishment of optimal conditions with a reduced number of experiments. The number of experiments required for the CCD optimization is determined by Eq. S3, where *k* is the number of experimental variables to be optimized, and *n* is the number of replicates of the central point experimental conditions established. Within this equation, the term 2^k corresponds to the experiments to evaluate the 1st order effects of the factorial design and *2k* corresponds to the experiments to evaluate the 2nd order effects. These experiments must be at a distance $\alpha = 2^{(k/4)}$ from the central value.

$$N = 2^k + 2k + n \qquad \textbf{(Eq. S3)}$$

Considering the results obtained in the screening step, two variables, the pH and volume of the aqueous phase, were optimized. A total of 11 experiments were performed, considering three replicates for the experiments at the central point level. The experimental values of the variables evaluated for the five levels of the CCD, coded as +1 (high level), 0 (central point), -1 (low level), and ±1.414 (distance $\alpha = 2^{(2/4)}$ from the central point), are shown in Table S2.

Table S2. Conditions of Central Composite Design of experiments and overall response (OR) obtained. Uncoded values selected for each variable are included in brackets.

Experiment	Variables				OR
	**V_1**		**V_6**		
1	+1	(7.1)	-1	(2.20)	0.51
2	-1	(2.9)	-1	(2.20)	0.69
3	+1	(7.1)	+1	(7.80)	0.56
4	-1	(2.9)	+1	(7.80)	0.66
5	0	(5.0)	+1.414	(9.00)	0.46
6	0	(5.0)	-1.414	(1.00)	0.64
7	+1.414	(8.0)	0	(5.00)	0.62
8	-1.414	(2.0)	0	(5.00)	0.72
9	0	(5.0)	0	(5.00)	0.74
10	0	(5.0)	0	(5.00)	0.72
11	0	(5.0)	0	(5.00)	0.77

V_1: pH of aqueous phase; **V_6**: Aqueous phase volume (mL)

From the OR obtained in the CCD experiments, the following second order polynomial equation was obtained using the least square method:

$$OR = 0.74 - 0.029\ V_1 - 0.055\ V_6 - 0.097\ V_1^2 - 0.038\ V_6^2 + 0.020\ V_1V_6 \quad \textbf{(Eq. S4)}$$

Where V_1 is the pH of the aqueous phase and V_6 is the volume of aqueous phase.

A Student *t-test* was performed to evaluate the significance of the coefficients of the mathematical model. The experimental *t*-student value was calculated using the Eq. S5, where $|b|$ is the absolute value of the coefficient, σ_{rep} the standard deviation of the central point replicates, and C_{jj} the diagonal term of the $[X^T \cdot X]^{-1}$matrix.

$$t_{exp} = \frac{|b|}{\sigma_{rep}\sqrt{C_{jj}}} \quad \textbf{(Eq. S5)}$$

The values of the coefficients of the mathematical model and the experimental *t*-student value calculated for each coefficient are shown in Table S3.

Table S3. Results of statical parameters for Student-t test of the fitted mathematical model.

Term	Coefficient	σ_{rep}	C_{jj}	t_{exp}
b_0	0.74	0.0282	0.250	62.4
b_1	-0.029	0.0282	0.125	3.46
b_6	-0.055	0.0282	0.125	6.50
b_{11}	-0.097	0.0282	0.094	13.3
b_{66}	-0.038	0.0282	0.094	5.18
b_{16}	0.020	0.0282	0.250	1.69

The critical t-student value for a 95% confidence level and 2 degrees of freedom is 4.30. As can be seen in Table S3, the experimental t-student value of the coefficients b_1 (3.46) and b_{16} (1.69) are lower than the critical t-student value, so these coefficients were considered non-significant and excluded from the polynomial equation. Therefore, the equation that describes the response of the system is as follows:

$$OR = 0.74 - 0.055\ V_6 - 0.097\ V_1^2 - 0.038\ V_6^2 \quad \textbf{(Eq. S6)}$$

The correlation between the experimental data and those obtained using the mathematical model of Eq. S6 was estimated. The linear correlation coefficient (*r*) of the correlation was 0.9220, indicating that an accurate response can be obtained with the predicted mathematical model. The reliability of the mathematical model was also evaluated using the Fisher test (Eq. S7), where σ_{res}^2 the residual variance of the model coefficients and σ_{rep}^2 *is* the variance of replicates obtained from the three center level experiments.

$$F = \frac{\sigma_{res}^2}{\sigma_{rep}^2} \quad \textbf{(Eq. S7)}$$

The experimental F value obtained (3.76) was lower than the critical F value for a 95% confidence level ($F_{7,2}$ = 19.35), indicating that there are non-significant differences between the variances and thus the mathematical model is adequate to predict the system response. From the obtained mathematical model (Eq. S6), the response surface was constructed (included in the manuscript).

SECTION S2. CALIBRATION AND ANALYTICAL FIGURES OF MERIT

Table S4. Instrumental UHPLC-QTOF-MS limits of detection (LODs) and limits of quantification (LOQs)

Analyte	Instrumental LOD / ng mL^{-1}	Instrumental LOQ / ng mL^{-1}
BTR	0.69	2.32
5Me-BTR	0.60	1.99
2OH-BTH	1.31	4.37
5,6diMe-BTR	0.99	3.30
2Am,6Cl-BTH	0.31	1.04

BP3	1.26	4.20
OCT	1.40	4.68
MP	0.95	2.86
PP	0.47	1.40

SECTION S3. SUSTAINABILITY, PRACTICALITY, AND APPLICABILITY EVALUATION

ChlorTox:

The formulas used to calculate the Weighted Number of Hazards (WNH) for each reagent and the total ChlorTox score are shown in Eq. S8 and Eq. S9. In these equations, N_{cat} is the number of hazards of a given risk category included in the safety data sheet (SDS) and m_{sub} is the mass of reagent consumed in the analysis. The WNH of chloroform (CH_{CHCl3}) was 5.75.

$$WHN(CH_{sub}) = 1N_{Cat_1} + 0.75N_{Cat_2} + 0.5N_{Cat_3} + 0.25N_{Cat_4} \qquad \textbf{(Eq. S8)}$$

$$ChlorTox = \sum \frac{CH_{sub}}{CH_{CHCl3}} * m_{sub} \qquad \textbf{(Eq. S9)}$$

The obtained values of WNH and the reagent masses used are included in Table S5.

Table S5. WNH values and used mass in a single analysis for each reagent.

Method	Reagent	WNH	CH_{sub} / CH_{CHCl3}	m_{sub} (g)
DLLME	Thymol	3.00	0.52	0.080
	Menthol	1.50	0.26	0.020
HPLC-QTOF	Methanol	3.25	0.57	12
	Ammonium formate	0.75	0.13	0.005
	Formic acid	3.25	0.57	0.002
	Ammonium acetate	0.00	0.00	0.005

AGREEprep and AGREE:

The criterion of AGREEprep and AGREE metrics and the answers provided to the software to evaluate the here developed method are summarized in Table S6.

Table S6. Criteria answers in AGREEprep and AGREE metrics

AGREEprep CRITERION	AGREE CRITERION
**1.** Sample preparation placement → *Ex-situ*	**1.** Sample treatment placement → Off-line
**2.** Hazardous materials → *0.005 g* [A]	**2.** Sample amount→ 5.00 mL
**3.** Sustainability-renewability of materials → *> 75%*	**3.** Device position→ Off-line
**4.** Waste → *5.00 mL*	**4.** Sample prep. stages→ 3 or fewer
**5.** Size economy of the sample → *5.00 mL*	**5.** Automation/minimization→ *Semiautomatic/ miniaturized*
**6.** Sample throughput →*25-30 samples/h*	**6.** Derivatization→ Non used
**7.** Integration and automation:	**7.** Waste→ ≈20 mL
a. Steps in the sample preparation → *2 steps*	**8.** Analysis throughput→ 9 analytes / 3 samples/h
b. Degree of automation → *Semiautomatic*	**9.** Energy consumption→ LC-MS [B]
**8.** Energy consumption → *Heating plate and centrifuge*	**10.** Source of reagents→Some biobased
**9.** Post-sample preparation analysis → *UHPLC-QTOF*	**11.** Toxicity→ 6 g [A]
**10.** Operator's safety →*no hazards or no exposure*	**12.** Operator´s safety→ 1 hazard (flammable)

[A] The ChlorTox mass values obtained were used ; [B] Only the instrumentation with the highest energy consumption has been specified

BAGI:

Table S7. Criteria answers in BAGI metric

BAGI CRITERION
**1.** Type of analysis → Quantitative and confirmatory
**2.** Multi- or single analyte determination → Multi- analysis (2-15 compounds of different families)
**3.** Analytical technique → Sophisticate instrumentation (UHPLC-QTOF)
**4.** Simultaneous sample preparation → 2-12
**5.** Sample pretreatment → Miniaturized extraction sample preparation (DLLME)
**6.** Samples analyses per hour→ 2-4
**7.** Reagents and materials → Common commercially available
**8.** Necessity of preconcentration→ Preconcentration required
**9.** Degree of automation → Semi-automated with common devices
**10.** Amount of sample → < 10 mL

SECTION S4. APPLICATION AND VALIDATION.

Table S8. Concentrations (mean ± standard deviation, n = 3) and recoveries found for the target analytes in the fortified wastewater 1 (WW-1).

Analyte	Add (ng mL^{-1})	Found (ng mL^{-1})	Recovery (%)
BTR	-	0.32±0.05	-
	0.204	0.43±0.04	82±8
5Me-BTR	-	0.35±0.02	-
	0.199	0.56±0.06	102±10
2OH-BTH	-	0.82±0.03	-
	0.201	0.91±0.07	89±7
5,6diMe-BTR	-	<0.048	-
	0.200	0.18±0.01	89±6
2Am,6Cl-BTH	-	<0.016	-
	0.204	0.18±0.02	88±11
BP3	-	0.57±0.03	-
	0.200	0.80±0.03	104±3
OCT	-	0.91±0.03	-
	0.201	1.1±0.1	96±10
MP	-	<0.088	-
	0.200	0.18±0.01	88±3
PP	-	<0.024	-
	0.186	0.20±0.01	106±6

SECTION S5. EXPERIMENTAL

Table S9. Chemical structures and properties of the analytes

BENZOTHIAZOLE (BTH)		
Analyte	***Chemical structure***	***Properties***
2-amino-6-chloro benzothiazole (2-am-6-Cl BTH)<br>CAS: 95-24-9	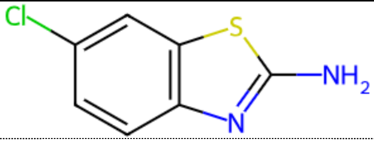	pKa = 1.2<br>$LogK_{ow}$ = 1.43
2-hydroxy-benzothiazole (2-OH BTH)<br>CAS: 934-34-9	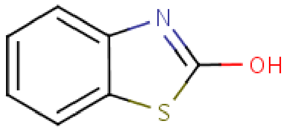	pKa = 7.8<br>$LogK_{ow}$ = 1.73

BENZOTRIAZOLE (BTR)		
Analyte	***Chemical structure***	***Properties***
1-H Benzotriazole (BTR)<br>CAS: 95-14-7	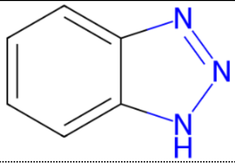	pKa = 9.8<br>$LogK_{ow}$ =1.21
5-methyl benzotriazole (5-Me-BTR)<br>CAS: 136-85-6	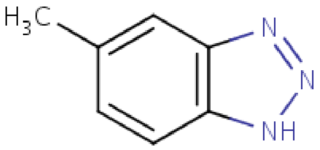	pKa = 10.1<br>$LogK_{ow}$ = 1.60
5,6 dimethyl benzotriazole (5,6 diMe-BTR)<br>CAS: 4184-79-6	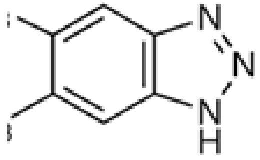	pKa = 10.3<br>$LogK_{ow}$ =2.06

UV FILTERS		
Analyte	***Chemical structure***	***Properties***
Octocrylene (Oct)<br>CAS: 6197-30-4	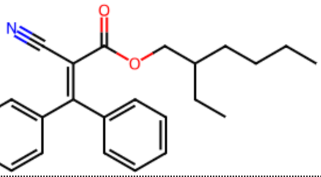	pKa = 10.3<br>$LogK_{ow}$ = 5.37
Benzophenone 3 (BP3)<br>CAS: 131-57-7	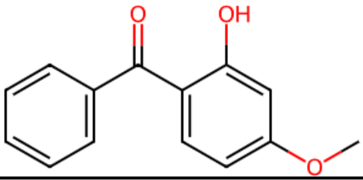	pKa = 7.1<br>$LogK_{ow}$ = 3.79

PARABENS		
Analyte	***Chemical structure***	***Properties***
Methylparaben (MP)<br>CAS: 99-76-3	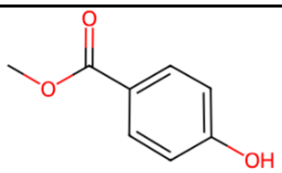	pKa = 8.17<br>$LogK_{ow}$ = 1.66
Propylparaben (PP)<br>CAS: 94-13-3	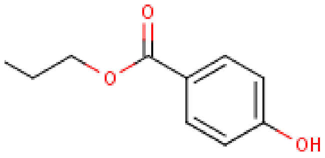	pKa = 8.35<br>$LogK_{ow}$ = 2.71

Table S10. UHPLC-QTOF-MS gradient elution program

Time (min)	Flow rate (mL min^{-1})	A (%)	B (%)
0 to 1	0.20	99.0	1.0
1 to 3	0.20	61.0	39.0
3 to 14	0.40	0.1	99.9
14 to 16	0.48	0.1	99.9
16 to 19	0.48	99.0	1.0
19 to 20	0.20	99.0	1.0
